# Infection by CXCR4-Tropic Human Immunodeficiency Virus Type 1 Is Inhibited by the Cationic Cell-Penetrating Peptide Derived from HIV-1 Tat

**DOI:** 10.1155/2012/349427

**Published:** 2012-01-29

**Authors:** Shawn Keogan, Shendra Passic, Fred C. Krebs

**Affiliations:** Department of Microbiology and Immunology, Center for Molecular Virology and Translational Neuroscience, and Center for Sexually Transmitted Disease, Institute for Molecular Medicine and Infectious Disease, Drexel University College of Medicine, Philadelphia, PA 19102, USA

## Abstract

Cell-penetrating peptides (CPP), which are short peptides that are capable of crossing the plasma membrane of a living cell, are under development as delivery vehicles for therapeutic agents that cannot themselves enter the cell. One well-studied CPP is the 10-amino acid peptide derived from the human immunodeficiency virus type 1 (HIV-1) Tat protein. In experiments to test the hypothesis that multiple cationic amino acids within Tat peptide confer antiviral activity against HIV-1, introduction of Tat peptide resulted in concentration-dependent inhibition of HIV-1 IIIB infection. Using Tat peptide variants containing arginine substitutions for two nonionic residues and two lysine residues, HIV-1 inhibition experiments demonstrated a direct relationship between cationic charge and antiviral potency. These studies of Tat peptide as an antiviral agent raise new questions about the role of Tat in HIV-1 replication and provide a starting point for the development of CPPs as novel HIV-1 inhibitors.

## 1. Introduction

Cell penetrating peptides (CPP) are short peptides that can efficiently cross the plasma membrane, which is otherwise a formidable barrier to many extracellular molecules [1–3]. CPPs are capable of not only traversing the cell membrane, but also serving as a vehicle for transporting a variety of cargos, including nucleic acids, polymers, nanoparticles, and drugs that cannot otherwise gain entry to the cell [3]. Although the functions of various CPPs have been repeatedly verified in a variety of cells and conditions, the mechanism of CPP uptake is not yet fully understood and may involve energy-dependent and -independent mechanisms [4].

Of the numerous peptides shown to have cell penetrating properties, a 10-amino acid (aa) peptide derived from the human immunodeficiency virus type 1 (HIV-1) Tat protein has been well studied as an effective CPP and an attractive drug delivery agent [5]. The Tat peptide has received particular emphasis as a CPP due to its simplicity and capacity for modification to suit the delivery context or cargo [5, 6]. The core peptide is a 10-aa sequence comprised of six arginine and two lysine residues, as well as two non-ionic amino acids (Table 1). However, numerous Tat peptides of varied lengths and terminal sequences have been investigated with the goals of modifying activity or attaching different cargo [6]. A multitude of studies have determined that the activity of the Tat peptide as a CPP involves interactions with the cellular membrane and cytoskeleton [7], and is influenced by numerous variables related to the peptide, the cargo, and extracellular conditions [4].

CPPs such as the Tat peptide, the 16-aa penetratin peptide derived from the Drosophila melanogaster Antennapedia homeodomain protein, and nona-arginine contain numerous cationic arginine (R) and lysine (K) residues [2]. Interestingly, cationic charge is a feature also shared by molecules identified as inhibitors of HIV-1 infection. Multiple cationic charges are prominent features of molecules shown to have activity against HIV-1, including ALX40-4C [8], NeoR6 (an aminoglycoside-arginine conjugate) [9], the lysozyme-derived HL9 peptide [10], the cathelicidin LL-37 [11], the biguanide-based molecule NB325 [12–16], and compounds that incorporate multiple guanide groups [17]. Cationic peptides found in both semen and cervicovaginal fluids were shown to effectively inhibit HIV-1 infection [18, 19]. Indeed, full-length HIV-1 Tat protein, from which the cationic Tat peptide was derived, was shown to inhibit HIV-1 infection as a CXCR4 antagonist [20].

The present studies were conducted to test the hypothesis that Tat peptide, because of the numerous cationic amino acids contained within its primary sequence, can effectively inhibit HIV-1 infection. In vitro experiments involving Tat peptide and an HIV-1-susceptible indicator cell line demonstrated concentration-dependent inhibition of the X4 HIV-1 strain IIIB, which uses CXCR4 as a coreceptor. Additional experiments involving variants of Tat peptide with increased cationic charge suggested a direct relationship between charge magnitude and antiviral potency. These results provide further insights into a potential role for Tat as an HIV-1 inhibitor and suggest a novel anti-HIV-1 activity attributed to the family of CPPs.

## 2. Materials and Methods

### 2.1. Synthesis of Tat Peptide and Variants

Tat peptide (Table 1) was derived from residues 48–57 (numbering from HIV-1 strain SF2) of the full-length Tat protein [5, 6, 21]. Three arginine-enriched Tat peptide variants (Table 1) were designed by substituting arginine for G48, Q54, or both amino acids. A decaarginine peptide was also included in these studies as a Tat peptide variant with all four nonarginine residues converted to arginines. All peptides were synthesized commercially by liquid phase peptide synthesis (GenScript, Pascataway, NJ) and provided at >95% purity as determined by mass spectrometry and high-performance liquid chromatography analysis performed by the manufacturer (GenScript). Lyophilized peptides were suspended in 1 mL of sterile deionized water upon receipt and stored at −20°C prior to use.

### 2.2. Cell Line Maintenance

P4-R5 MAGI indicator cells (NIH AIDS Research and Reference Reagent Program number 3580) were maintained in Dulbecco's modified eagle's media (DMEM) supplemented with 10% fetal bovine serum (FBS), 0.05% sodium bicarbonate, antibiotics (penicillin, streptomycin, and kanamycin at 40 *μ*g/mL), and 1 *μ*g/mL puromycin (Cellgro, Manassas, VA).

### 2.3. Assessing Inhibition of HIV-1 Infection by Tat Peptide and Its Variants

Peptide effectiveness was determined in an HIV-1 infection inhibition assay using P4-R5 MAGI indicator cells. P4-R5 MAGI cells were plated at a concentration of 1.5 × 10^4^ cells/well in a flat-bottom 96-well plate (BD Biosciences, Bedford, MA). The cells were then infected with HIV-1 strain IIIB (Advanced Biotechnologies, Inc., Columbia, MD; 10^7.8^ TCID_50_/mL) at multiplicities of infection (MOI) of 0.6, 0.05, or 0.03 in the presence or absence of peptide or dextran sulfate (DS) (Sigma, St. Louis, MO). Following a 2 h incubation at 37°C, the cells were washed with PBS, provided with 200 *μ*L of new media, and incubated for an additional 46 h. Levels of infection were measured using the Galacto-Star-*β*-Galactosidase Reporter Gene Assay System for Mammalian Cells (Applied Biosystems, Carlsbad, CA) as described by the manufacturer. Chemiluminescence was measured using a Glomax Luminometer plate reader (Promega, Madison, WI).

### 2.4. Assessing the Effect of Tat Peptide on In Vitro Cell Viability

P4-R5 MAGI cells were plated at a concentration of 1.5 × 10^4^ cells/well in a flat-bottom 96-well plate. The cells were then exposed to the indicated half log concentrations of peptide and incubated at 37°C for 2 h. Following exposure, the cells were washed with PBS and then assayed for cell viability immediately or at 24 h or 48 h after exposure. Viability was measured using a 3-(4,5-dimethylthiazol-2-yl)-2,5-diphenyltetrazolium bromide (MTT) assay as previously described [13].

### 2.5. Data Analyses

Mean values and standard deviations were calculated from two independent assays in which each concentration was examined in quadruplicate. Calculations of EC_50_ (concentrations that resulted in 50% reductions in infection relative to mock-treated, HIV-1-infected cells) were calculated using the Forecast function of Microsoft Excel.

## 3. Results

### 3.1. Tat Peptide Inhibits HIV-1 Infection

**Figure 1:**
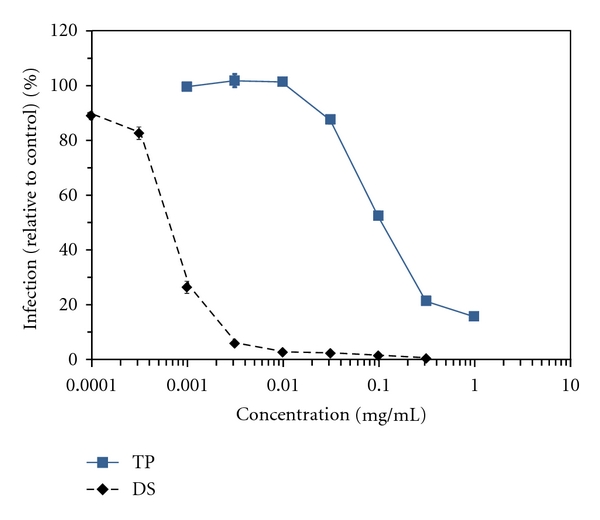
Tat peptide inhibits infection by HIV-1 strain IIIB. P4-R5 MAGI cells were exposed to half log concentrations of Tat peptide (TP) or dextran sulfate (DS) in the presence of HIV-1 strain IIIB for 2 h. Reductions in HIV-1 infection (%) were calculated relative to mock-exposed HIV-1 infected cells. The graph represents data from two independent assays in which infections at each concentration were repeated in quadruplicate. Error bars represent standard deviations.

Initial experiments were performed to test the hypothesis that Tat peptide, by virtue of its cationic charge, was capable of inhibiting HIV-1 infection. HIV-1-susceptible P4-R5 MAGI indicator cells were exposed to HIV-1 strain IIIB (0.6 MOI) for 2 h while in the presence of half log concentrations of Tat peptide up to 1 mg/mL. The presence of Tat peptide inhibited HIV-1 infection in a concentration-dependent manner (Figure 1), with an EC_50_ of 0.094 mg/mL (50 *μ*M). No inhibition was apparent at or below 0.0316 mg/mL. At the highest concentration tested (1 mg/mL), the presence of Tat peptide was insufficient to completely inhibit HIV-1 infection (15.6% infection relative to mock-exposed, HIV-1-infected cells). In comparison, the anionic compound dextran sulfate, which was included as a known inhibitor, blocked HIV-1 infection with an EC_50_ of 0.0007 mg/mL. To determine the potential effect of virus titer on Tat peptide antiviral activity, similar experiments were performed using reduced concentrations of input virus (0.05 and 0.03 MOI). In these experiments (Table 2), reductions in input virus had no effect on Tat peptide antiviral activity (EC_50_ values of 0.14 mg/mL and 0.10 mg/mL, resp.).

**Figure 2:**
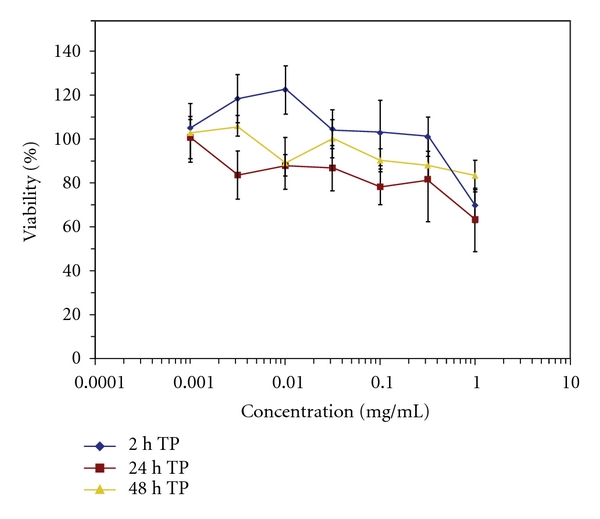
Tat peptide has no effect on reporter cell viability. P4-R5 MAGI cells were exposed to half log concentrations of Tat peptide for 2 h, washed, and assessed immediately for changes in cell viability or after extended maintenance (24 h or 48 h after exposure) in the absence of Tat peptide. Percent changes in cell viability were calculated relative to mock-exposed cells. The graph represents data from two independent assays in which exposure to each concentration of peptide was repeated in quadruplicate. Error bars represent standard deviations.

To confirm that any adverse effects of Tat peptide on reporter cell viability had not compromised the antiviral assays, MTT cytotoxicity assays were performed using conditions identical to those used in the antiviral assays. In these assays, 2 h exposures to Tat peptide at concentrations below 1 mg/mL had no effect on P4-R5 MAGI cell viability, as measured immediately after exposure or after extended postexposure maintenance (24 h or 48 h) in the absence of Tat peptide (Figure 2). These results indicated that measurements of antiviral activity were not biased by reductions in P4-R5 MAGI cell viability. These results are also consistent with previous studies [22], in which Tat peptide alone (but not peptide conjugated to payload) had no effect on cell viability at concentrations up to 100 *μ*M and exposure durations as long as 48 h.

### 3.2. Additional Cationic Charges Increase the Antiviral Potency of Tat Peptide

 Having demonstrated the anti-HIV-1 activity of the Tat peptide, additional experiments were performed to investigate the role of charge in determining antiviral efficacy. Of the 10 aa residues in Tat peptide, eight are cationic (six arginine and two lysine residues) and the remaining two are uncharged (G48, nonpolar and aliphatic; Q54, polar). To increase the net peptide charge, arginine residues were substituted for one or both of the noncationic residues in the native Tat peptide sequence (Table 1). These substitutions increased the net positive side chain charge of the Tat peptide from +8 to +9 (TPvar1 and TPvar2) or +10 (TPvar3). An additional peptide, decaarginine (R-10), was also included in these studies. R-10 also had a net side chain charge of +10, but differed from TPvar3 in that all ten positive charges were contributed by the arginine guanidinium groups. R-10 was, in effect, a Tat peptide variant with arginine residues substituted into all nonarginine positions. Like the Tat peptide, none of the variants had any effect on P4-R5 MAGI cell viability after a 2 h exposure (data not shown).

**Figure 3:**
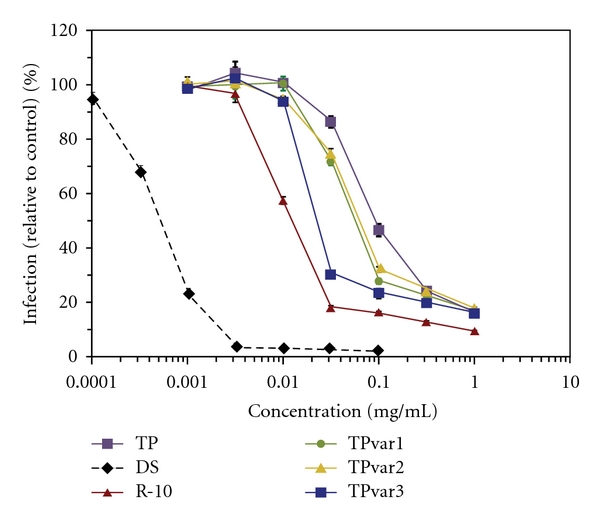
Increased peptide antiviral potency is associated with increased peptide cationic charge. P4-R5 MAGI cells were exposed to half log concentrations of Tat peptide (TP), three Tat peptide variants (TPvar1-3), decaarginine (R-10), or dextran sulfate (DS) in the presence of HIV-1 strain IIIB for 2 h. Peptide sequences are depicted in Table 1. Reductions in HIV-1 infection (%) were calculated relative to mock-exposed HIV-1 infected cells. The graph represents data from two independent assays in which infections at each concentration were repeated in quadruplicate. Error bars represent standard deviations.

Concurrent incubation of HIV-1 IIIB and each peptide with P4-R5 MAGI cells again resulted in concentration-dependent inhibition of HIV-1 infection (Figure 3). However, the Tat peptide, the Tat peptide variants, and R-10 differed in antiviral potency, with EC_50_ values ranging from 0.094 mg/mL (Tat peptide) to 0.014 mg/mL (R-10). Single substitutions of arginine into the Tat peptide sequence (G48R or Q54R) resulted in small increases in antiviral activity relative to Tat peptide (TPvar1 EC_50_ = 0.065 mg/mL; TPvar2 EC_50_ = 0.071 mg/mL). Arginine substitutions at both positions (TPvar3) further increased peptide antiviral activity (EC_50_ = 0.025 mg/mL). However, despite having the same number of positive charges, TPvar3 was less active than R-10. No peptide was active below 0.00316 mg/mL or 100% inhibitory at the highest concentration examined (1 mg/mL).

## 4. Discussion

Results presented in this paper demonstrate that Tat peptide, a CPP that is capable of delivering molecules intracellularly [1, 5] across living membranes, also contains intrinsic antiviral activity against HIV-1 infection. Nontoxic concentrations of Tat peptide inhibited CXCR4-mediated infection by the X4 virus HIV-1 IIIB in a concentration-dependent manner. In experiments designed to explore the contribution of cationic charge to antiviral activity, Tat peptide variants with arginine substitutions for non-ionic and lysine residues were also assessed for antiviral activity. Increases in antiviral potency with increased net peptide positive charge confirmed our original hypothesis and suggest a role for peptide charge in the mechanism of action against HIV-1 infection.

In a broader virologic context, observations reported herein provide new information about Tat protein and its contributions to HIV-1-associated pathogenesis. In previous studies, soluble full-length Tat protein, which is secreted from infected cells [23], specifically inhibited an HIV-1 strain that used CXCR4 as a co-receptor (designated X4) but not a CCR5-tropic (R5) strain [20]. Our preliminary results using the Tat peptide are consistent with this observation (data not shown). The authors speculated that, during the course of HIV-1 infection, this mechanism attributed to extracellular Tat could favor the replication and spread of R5 virus by inhibiting X4 virus infection. They further suggested that Tat protein may interact with acidic regions of CXCR4 through the high concentration of basic residues scattered throughout the Tat protein primary sequence [20]. However, this study did not identify the specific sequences within full-length Tat that were the source of the antiviral activity. This antiviral activity was also complicated by apparent cytotoxicity associated with exposure to extracellular full-length Tat protein [24–27]. In contrast, the present studies demonstrated that the Tat peptide had no adverse effect on cell viability.

These studies also provide two starting points for the development of novel inhibitors of HIV-1. First, Tat peptide can serve as a prototype for the development of novel agents effective against HIV-1. Such agents may take the form of cationic peptides or small molecule inhibitors that mimic a peptide structure. Second, Tat peptide provides the basis for multifunctional therapeutic agents that combine the intrinsic and specific anti-HIV-1 activity of Tat peptide with its ability to deliver therapeutic agents that by themselves do not readily penetrate cells and tissues [3, 6]. For example, Tat peptide could be linked to an HIV-1 protease inhibitor to form a dual-activity antiretroviral agent that combines entry inhibition, increased drug penetration, and a second, distinct mechanism of antiretroviral activity.

Our experiments also indicate that this intrinsic antiviral activity is not limited to the Tat-derived CPP alone. In the present studies, the R-10 peptide was also an effective HIV-1 inhibitor, despite changes in four out of ten amino acids with respect to the Tat peptide. Preliminary studies have also demonstrated anti-HIV-1 activity associated with the well-studied CPP nona-arginine (R-9) and a 20-aa peptide consisting solely of alternating arginine and glycine residues (data not shown). The finding that antiviral activity is not limited to Tat peptide suggests that a key characteristic common to these molecules (i.e., multiple cationic charges) confers activity against HIV-1.

The involvement of cationic charge in CPP antiviral activity is also supported by the results of experiments involving the Tat peptide variants. Those results indicated a direct relationship between charge and antiviral activity. Tat peptide (+8 charge) was the least active while R-10 (+10 charge) was the most active, and variants with intermediate levels of cationic charge had intermediate levels of antiviral activity. Despite the fact that R-10 and TPvar3 had the same charge, these two peptides differed in their effects on HIV-1 infection, likely due to the replacement of two lysine residues with two arginine residues. Lysine has a single positive charge associated with a terminal amino group while arginine has a single positive charge associated with a terminal guanidinium group. The charge in arginine is delocalized across the guanidinium group, supporting the formation of multiple hydrogen bonds [2, 28]. Polar and charged interactions supported by the arginine end group may favor mechanisms that are responsible for antiviral activity and, perhaps, cell penetrating activity [5, 28]. Interestingly, peptides with multiple guanidinium groups, such as R-10 and nona-arginine, are in the same family with other demonstrated HIV-1 inhibitors ALX40-4C, NB325, and LL-37, which are also cationic molecules with multiple guanidinium groups.

Related studies have also indicated the importance of charge in cationic HIV-1 inhibitors and provided further evidence for a mechanism of CPP antiviral activity. We previously demonstrated that charge distribution plays a key role in the antiviral activity of biguanide-based molecules [13]. These molecules are oligomeric, cationic compounds characterized by the presence of alternating biguanide groups and hydrocarbon linkers [15]. Using rational compound design and structure-function screening of biguanide-containing synthetic molecules, studies identified polyethylene hexamethylene biguanide (PEHMB; also known as NB325) as a molecule with minimal cytotoxicity and considerable activity against HIV-1 [12, 15]. More recent work identified NB325 as an HIV-1 entry inhibitor [14, 15] that antagonizes CXCR4 through epitope-specific interactions with extracellular loop 2 (ECL2). Further experiments demonstrated relationships between charge density, cytotoxicity, and antiviral activity [13]. These findings add to a collective understanding of cationic HIV-1 inhibitors and can be used to guide further investigations focused on defining mechanisms of action and optimizing the antiviral potency of cationic inhibitors such as the CPPs.

These results provide the basis for further basic science and translational studies. Expanded studies will be necessary to investigate the antiviral effect of Tat and Tat peptide on HIV-1 replication in natively HIV-1-susceptible immune cell populations and to better understand the contribution of the potential bias toward R5 virus replication to viral pathogenesis and disease progression. Related efforts will be directed toward the development of novel CPP-based antiviral agents that can serve as multifunctional HIV-1 inhibitors. These efforts will address CPP potency, stability, mechanism of action, and combined activity as these agents are advanced into preclinical investigations and clinical trials.

## Figures and Tables

**Table 1:** Sequences of peptides examined. Peptide sequences are shown relative to the primary amino acid sequence of the Tat peptide. Position numbers are derived from the full-length Tat protein amino acid sequence (HIV-1 strain SF2) [21].

Peptide	Sequence										Charge
Tat peptide	G	R	K	K	R	R	Q	R	R	R	+8
TPvar1	R	—	—	—	—	—	—	—	—	—	+9
TPvar2	—	—	—	—	—	—	R	—	—	—	+9
TPvar3	R	—	—	—	—	—	R	—	—	—	+10
R-10	R	—	R	R	—	—	R	—	—	—	+10

aa position	48	49	50	51	52	53	54	55	56	57	

**Table 2:** Viral titer does not affect the antiviral activity of Tat peptide. EC_50_ values were calculated from the results of antiviral assays (as described in Section 2) involving infection by HIV-1 IIIB at three different multiplicities of infection (MOI).

Virus concentration during infection (10^3^ infectious virions/mL)	MOI	EC_50_
88	0.6	0.094 mg/mL
8.8	0.05	0.14 mg/mL
4.4	0.03	0.10 mg/mL

## References

[1] Foerg C, Weller KM, Rechsteiner H (2008). Metabolic cleavage and translocation efficiency of selected cell penetrating peptides: a comparative study with epithelial cell cultures. *American Association of Pharmaceutical Scientists Journal*.

[2] Nakase I, Takeuchi T, Tanaka G, Futaki S (2008). Methodological and cellular aspects that govern the internalization mechanisms of arginine-rich cell-penetrating peptides. *Advanced Drug Delivery Reviews*.

[3] Torchilin VP (2008). Tat peptide-mediated intracellular delivery of pharmaceutical nanocarriers. *Advanced Drug Delivery Reviews*.

[4] Jones AT (2008). Gateways and tools for drug delivery: endocytic pathways and the cellular dynamics of cell penetrating peptides. *International Journal of Pharmaceutics*.

[5] Gump JM, Dowdy SF (2007). TAT transduction: the molecular mechanism and therapeutic prospects. *Trends in Molecular Medicine*.

[6] Brooks H, Lebleu B, Vivès E (2005). Tat peptide-mediated cellular delivery: back to basics. *Advanced Drug Delivery Reviews*.

[7] Mishra A, Lai GH, Schmidt NW (2011). Translocation of HIV TAT peptide and analogues induced by multiplexed membrane and cytoskeletal interactions. *Proceedings of the National Academy of Sciences of the United States of America*.

[8] Doranz BJ, Grovit-Ferbas K, Sharron MP (1997). A small-molecule inhibitor directed against the chemokine receptor CXCR4 prevents its use as an HIV-1 coreceptor. *Journal of Experimental Medicine*.

[9] Lapidot A, Peled A, Berchanski A (2008). NeoR6 inhibits HIV-1-CXCR4 interaction without affecting CXCL12 chemotaxis activity. *Biochimica et Biophysica Acta*.

[10] Lee-Huang S, Maiorov V, Huang PL (2005). Structural and functional modeling of human lysozyme reveals a unique nonapeptide, HL9, with anti-HIV activity. *Biochemistry*.

[11] Bergman P, Walter-Jallow L, Broliden K, Agerberth B, Söderlund J (2007). The antimicrobial peptide LL-37 inhibits HIV-1 replication. *Current HIV Research*.

[12] Krebs FC, Miller SR, Ferguson ML, Labib M, Rando RF, Wigdahl B (2005). Polybiguanides, particularly polyethylene hexamethylene biguanide, have activity against human immunodeficiency virus type 1. *Biomedicine and Pharmacotherapy*.

[13] Passic SR, Ferguson ML, Catalone BJ (2010). Structure-activity relationships of polybiguanides with activity against human immunodeficiency virus type 1. *Biomedicine and Pharmacotherapy*.

[14] Thakkar N, Pirrone V, Passic S (2010). Persistent interactions between biguanide-based compound NB325 and CXCR4 result in prolonged inhibition of human immunodeficiency virus type 1 infection. *Antimicrobial Agents and Chemotherapy*.

[15] Thakkar N, Pirrone V, Passic S (2009). Specific interactions between the viral coreceptor CXCR4 and the biguanide-based compound NB325 mediate inhibition of human immunodeficiency virus type 1 infection. *Antimicrobial Agents and Chemotherapy*.

[16] Lozenski K, Kish-Catalone T, Pirrone V (2011). Cervicovaginal safety of the formulated biguanide-based human immunodeficiency virus type 1 (HIV-1) inhibitor NB325 in a murine model of microbicide application. *Journal of Biomedicine and Biotechnology*.

[17] Wilkinson RA, Pincus SH, Shepard JB (2011). Novel compounds containing multiple guanide groups that bind the HIV coreceptor CXCR4. *Antimicrobial Agents and Chemotherapy*.

[18] Martellini JA, Cole AL, Venkataraman N (2009). Cationic polypeptides contribute to the anti-HIV-1 activity of human seminal plasma. *FASEB Journal*.

[19] Venkataraman N, Cole AL, Svoboda P, Pohl J, Cole AM (2005). Cationic polypeptides are required for anti-HIV-1 activity of human vaginal fluid. *Journal of Immunology*.

[20] Xiao H, Neuveut C, Tiffany HL (2000). Selective CXCR4 antagonism by Tat: implications for in vivo expansion of coreceptor use by HIV-1. *Proceedings of the National Academy of Sciences of the United States of America*.

[21] Kuppuswamy M, Subramanian T, Srinivasan A, Chinnadurai G (1989). Multiple functional domains of Tat, the trans-activator of HIV-1, defined by mutational analysis. *Nucleic Acids Research*.

[22] Cardozo AK, Buchillier V, Mathieu M (2007). Cell-permeable peptides induce dose- and length-dependent cytotoxic effects. *Biochimica et Biophysica Acta*.

[23] Ensoli B, Barillari G, Salahuddin SZ, Gallo RC, Wong-Staal F (1990). Tat protein of HIV-1 stimulates growth of cells derived from Kaposi’s sarcoma lesions of AIDS patients. *Nature*.

[24] Nath A, Psooy K, Martin C (1996). Identification of a human immunodeficiency virus type 1 Tat epitope that is neuroexcitatory and neurotoxic. *Journal of Virology*.

[25] Campbell GR, Pasquier E, Watkins J (2004). The glutamine-rich region of the HIV-1 Tat protein is involved in T-cell apoptosis. *Journal of Biological Chemistry*.

[26] Chauhan A, Tikoo A, Kapur AK, Singh M (2007). The taming of the cell penetrating domain of the HIV Tat: myths and realities. *Journal of Controlled Release*.

[27] Romani B, Engelbrecht S, Glashoff RH (2010). Functions of Tat: the versatile protein of human immunodeficiency virus type 1. *Journal of General Virology*.

[28] Rothbard JB, Jessop TC, Lewis RS, Murray BA, Wender PA (2004). Role of membrane potential and hydrogen bonding in the mechanism of translocation of guanidinium-rich peptides into cells. *Journal of the American Chemical Society*.

